# Artificial vagina conformation and composition for semen collection

**DOI:** 10.1530/RAF-25-0061

**Published:** 2025-12-03

**Authors:** Jacquline Rich, Dara N Orbach

**Affiliations:** Department of Life Sciences, Texas A&M University – Corpus Christi, Corpus Christi, Texas, USA

**Keywords:** artificial vagina, breeding, internal fertilizer, semen collection, vertebrate

## Abstract

**Abstract:**

Artificial vaginas (AVs) are tools used to collect semen from domestic and exotic species for artificial insemination and managed breeding programs. AVs differ widely in their shape, size, component parts, and material composition to fit species-specific applications. The quality of semen collected with AVs is inconsistent within and across species. This review examines peer-reviewed published literature on features of AVs used on non-human vertebrate taxa and assesses AV composition changes over time. Google Scholar, PubMed, and Web of Science databases were used to search for the term ‘AV’ from 1914 to 2024. After screening the abstracts and methods sections, 778 articles were included in the analysis. AVs were categorized based on AV material, shape, elasticity, and design augmentations. Over the past century, perissodactyls and artiodactyls have been the most studied orders for AV semen collection. Most AVs are made of rubber/latex or plastic, tube-shaped, and have a rigid outer shell and semi-elastic inner liner made of rubber/latex. AV materials, shapes, and elasticity have not changed substantially over time. Most commercially available AVs do not differ substantially from AVs developed originally for use with bulls and stallions, with few examples of species-specific AV compositions. As AVs are an increasingly important tool for semen collection, particularly among exotic species, future studies could consider how to reflect the genital variation of taxa within the shape, material, and design augmentations of AVs.

**Lay summary:**

AVs are devices used to collect semen from trained animals in human care for breeding programs. AVs have been developed for many bird and mammalian species. We examined the published literature to assess how AV compositions have changed across species since their initial use in 1914. We extracted data from the literature on AV materials, shapes, elasticities, and unique design features. Most AVs consisted of semi-elastic tube-shaped devices constructed of multiple materials. The most common design feature was a warmed bladder. AV compositions have not changed substantially over time or species, indicating opportunities for innovations such as incorporating anatomical features to improve semen quality.

## Introduction

Semen collection is increasingly important for breeding programs of domestic and exotic species (Bruno 2022). Semen collection followed by artificial insemination (AI) across managed breeding populations maintains genetic variation (Ballou *et al.* 2022). Despite advances in assisted reproductive technologies (ARTs) over the past several decades, AI success rates vary within and between domestic and exotic species (Howard & Wildt 2009, Amann & DeJarnette 2012, Faigl *et al.* 2012, Lonergan 2018). AI success has been improved by modifications to semen collection, processing, and preservation techniques, with research efforts particularly focused on developing species-specific protocols to preserve semen quality (Dziekońska & Partyka 2022).

Semen quality analyses historically focused on macroscopic ejaculate characteristics (e.g., color and volume) and subjective (not computer-aided) characterization of total sperm motility and concentration (Suárez *et al.* 2018). Technological advances have enabled multi-parameter assessments of semen quality, including objective analyses of sperm motility and kinematics, morphology and morphometrics, plasma membrane integrity, acrosome integrity, and DNA integrity using flow cytometry and computer-aided software (Graham 2001, Gillan *et al.* 2005, Paston *et al.* 2009, Michos *et al.* 2013, Bucher *et al.* 2019, Dolník *et al.* 2019, Tanga *et al.* 2021, Baldán *et al.* 2025). Semen quality analyses extend to biochemical and molecular components of sperm and seminal plasma (Caballero *et al.* 2012). Flow cytometry and microfluidic devices facilitate functional assessment of semen quality in environments that simulate the female reproductive tract (Leung *et al.* 2022, Vasilescu *et al.* 2023). Thresholds for high semen quality determination are species-specific, and methods for preserving semen vary (Shipley 1999, Kastelic & Thundathil 2008, Borate & Meshram 2022). While semen quality can be maintained over time by fine-tuning cryopreservation protocols (Borate & Meshram 2022), semen quality can also be improved at collection depending on the technique applied.

Semen is collected using a variety of tools and techniques that each present a unique set of benefits and drawbacks. One of the oldest sperm collection techniques entails sperm removal from the vas deferens of post-mortem individuals (Amann *et al.* 1974). Sperm collection from post-mortem males has been essential to obtain and preserve sperm from genetically modified laboratory species for biomedical research (Hart-Johnson & Mankelow 2022) and cryptic exotic species; however, the quality of sperm collected is inconsistent (e.g. 38.3% total sperm motility in tiger (*Panthera tigris*), 86.2% total sperm motility in springbok (*Antidorcas marsupialis*); Huijsmans *et al.* 2023) and decreases exponentially over time (Mennick 2023). Semen can be obtained from live animals by pharmaceutically induced ejaculation, commonly used with stallions and donkeys for which physiologically induced ejaculation is inhibited (Khanam *et al.* 2021). Surgical sperm extraction from the cauda epididymis under anesthesia, or microsurgical aspiration of the epididymal tubules, is commonly used for sperm collection from small domestic mammals such as rodents, canines, and felines (Boersma *et al.* 2015, Hassan *et al.* 2021, Anciuti *et al.* 2025). Pharmacological ejaculation with urethral catheterization has facilitated successful semen collection from exotic rodents and felines (Anciuti *et al.* 2025). Alternative collection techniques are preferred for larger domestic and exotic animal species, such as manual stimulation, electroejaculation, and artificial vaginas (AVs).

Manual stimulation, also known as the gloved hand technique, is a semen collection procedure used on many domestic and several captive exotic species (Crump & Crump 1989). Species-specific techniques include tactile stimulation of the abdominal, genital, and/or rectal region to induce ejaculation (Crump & Crump 1989). Manual stimulation is beneficial due to its simplicity, but has not been successful for all species. For example, manual stimulation of Muscovy drakes (*Cairina moschata*) elicited an aggressive response, which can endanger both the animals and their handlers (Gvaryahu *et al.* 1984). Within species where manual stimulation did not elicit a negative behavioral response, such as the greater one-horned rhinoceros (*Rhinoceros unicornis*), manual stimulation produced poorer quality semen samples than AV collection (Schaffer *et al.* 1990). Collection techniques that utilize specialized equipment often produce high-quality semen samples while minimizing risks associated with collection (Love 1992).

Beginning in the 1950s, electroejaculation (EEJ) became a popular semen collection technique (Dziuk *et al.* 1954). EEJ became particularly prevalent among species for which manual stimulation was challenging or prohibitive, such as cattle (*Bos taurus*) and non-domestic felids (Dziuk *et al.* 1954, Ackermann & Lopes 2020), although EEJ was also utilized on small laboratory animals such as rodents (Anciuti *et al.* 2025). Ejaculation is induced during EEJ by stimulating the prostate with a low-level electrical pulse administered by a rectal probe (Abraham *et al.* 2017). Semen quality from EEJ varies, yielding high-quality semen from cattle (Austin *et al.* 1961) and low-quality semen from dromedary camels (*Camelus dromedarius*; Al-Eknah *et al.* 2001) and sheep (*Ovis aries*; Aral & Aral 2004). To avoid injury to the animal, EEJ often necessitates anesthesia and requires specialized equipment that must be properly operated (Ackermann & Lopes 2020). Although EEJ has been opposed in some countries due to animal welfare concerns (Mujitaba *et al.* 2022), EEJ is a particularly important semen collection technique for individuals or species that are unwilling or unable to successfully use an AV (Clarke *et al.* 1973).

AVs are devices into which males ejaculate that became widely adopted in the early 1900s for semen collection from livestock species (Cole & Winters 1939). AVs are created to simulate the experience of copulation while reducing the disease transfer and ejaculate contamination that may occur during natural copulation (Cole & Winters 1939). AVs have been developed by international manufacturers (e.g., IMV Technologies, France and Minitube, Germany) and individual researchers (e.g. Harrop 1954) for use with domestic (e.g. cows (*Bos taurus*), (Cole & Winters 1939); horses (*Equus ferus caballus*), (Love 1992); and pigs (*Sus scrofa domesticus*), (Aamdal *et al.* 1958)) and exotic species (e.g. chimpanzee (*Pan troglodytes*); (Gould *et al.* 1993)). AVs differ in shape, size, and material based on the penis type (e.g. fibroelastic and musculocavernous) and ejaculatory mechanisms of the target species. While AVs can be handheld (e.g. Ackermann & Lopes 2020), the use of a dummy mount with attached AV (e.g. Aamdal *et al.* 1958) or intravaginal AV placed in a live female animal (e.g. Mansour 2023) reduces the proximity and duration of animal handling during semen collection. Although AV use requires animal training that may not be possible for all individuals or species (Wulster-Radcliffe *et al.* 2001), AVs often yield semen samples with higher motility (Aral & Aral 2004), acrosome integrity (Memon *et al.* 1986), and viability (Matthews *et al.* 2003) compared to semen samples collected by EEJ. AVs also yield semen samples with higher concentration (Sylla *et al.* 2015), motility (Kasai *et al.* 2001), and viability (Kasai *et al.* 2001) compared to semen samples collected by manual stimulation. The quality of semen collected using AVs is not consistent within or across species (Austin *et al.* 1961, Ledesma *et al.* 2014, Ledesma *et al.* 2015). As semen quality impacts AI success (Said & Land 2011), it is imperative to consider which AV features contribute to collection of high-quality semen. The goal of this review is to assess how AVs have changed in material, shape, elasticity, and design augmentations over time across non-human vertebrate species.

## Materials and methods

Google Scholar, PubMed, and Web of Science databases were used to search for the term ‘artificial vagina’ in articles published between January 1st 1914 and August 14th 2024. The year 1914 corresponds with the earliest described AV for a non-human vertebrate (dog (*Canis lupus familiaris*); Amantea 1914, Foote 2002). The abstract and methods sections of each article were reviewed and publications were excluded if they did not describe the use of an AV device, only related to invertebrates and/or externally fertilizing species, or exclusively discussed humans. Duplicate articles, news items, meeting abstracts, grants, notes, letters, patents, and retracted publications were also excluded. Review methods followed Prospero guidelines; however, submission to Prospero was inhibited as review content is not relevant to human health.

The methods and results sections in each remaining article were reviewed to categorize the AVs described based on their composition material(s), shape, elasticity, design augmentation(s), liner material, collection cone material, internal temperature, internal pressure, duration of contact, lubricant, and AV manufacturer and model, as available (Supplementary Table 1 (see section on Supplementary materials given at the end of the article)). Materials were categorized as glass, metal, mixed materials (for AVs constructed with multiple materials), plastic, or rubber. Shapes were categorized as biological system (for AVs that mimic species-specific morphology of the vagina), tube, or unformed/bag. Elasticities were categorized as flexible (all AV components could expand or contract), semi-elastic (some but not all AV components could expand or contract), or rigid (no AV components could expand or contract). Categories within design augmentations included air bladder, gel filter, imitation cervix, none (for AVs with no design augmentations), other, and warmed bladder. Collection cone shape was categorized as symmetrical, asymmetrical, or without specific shape. Liner material, collection cone material, internal temperature, internal pressure, duration of contact, lubricant use, and AV manufacturer and model were recorded when available. Photographs and descriptions of AVs were used to inform assignments within each category, as available. When information pertinent to specific categories (e.g. elasticity) was not explicitly stated in the article, contextual evidence from the AV descriptions/photographs was used for categorical assignment. When AVs could not be categorized using available or contextual information, the associated articles were excluded from the analyses. The study species and article publication year were recorded to explore taxonomic patterns and temporal changes.

Descriptive statistics were generated for each AV category in Microsoft Excel (v16.86). R statistical software (v4.2.3; R Core Team 2023) was used to generate data visualizations and develop a multinomial logistic regression model to measure temporal trends in AV categories using the nnet package (Venables & Ripley 2002). The Box-Tidwell test was used to test the assumption of linearity between time and the logit transformation of each dependent variable. Publication year was the independent variable and AV categories (material, shape, elasticity, and design augmentations) were the dependent variables. The feature comprising the highest percentage within an AV category was set as the baseline value (materials: mixed materials; shapes: tube; elasticities: semi-elastic; design augmentations: warmed bladder). Likelihood ratio tests were calculated to assess model significance using the MASS package (Venables & Ripley 2002).

## Results and discussion

The Google Scholar, PubMed, and Web of Science searches yielded 18,871 publications. Of these, 9,055 publications were removed from the analyses as they did not fit the inclusion criteria or were duplicate articles, 1,097 were excluded because there was no available English translation, 1,875 were removed because they did not describe nor cite the AV used, and 6,163 were removed because no online nor print version of the article could be accessed by the authors. The majority of studies that did not cite nor describe the use of an AV were focused on artiodactyls (48%, even-toed ungulates), followed by perissodactyls (35%, odd-toed ungulates). The remaining 778 articles were included in the analyses. The general trend of AV use on non-human vertebrates indicates changes among AV constructions, but a lack of significant changes in AV compositions over the past century.

### AV studies across species

**Figure 1:**
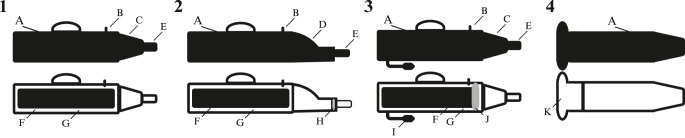
Schematic images of external (upper) and cross-section (lower) views of artificial vagina (AV) compositions, highlighting: 1) typical bovine AV/Hannover AV/Colorado AV, 2) Missouri AV, 3) typical bovine AV with multiple species-specific design augmentations, and 4) rigid, tube-shaped AV with no design augmentations and no collection cone. The component parts of AVs include: (A) AV body (with handle in models 1–3), (B) water/air filling valve, (C) symmetrical, cone-shaped collection cone, (D) asymmetrical collection cone, (E) semen collection vessel, (F) inner AV liner, (G) space between AV body and inner liner for air/water, (H) gel filter, (I) external air pump, (J) imitation cervix, and (K) soft material attached to semen collection vessel that directly contacts the penis (constructed of pipette bulb or similar material).

The earliest described AV was developed for domestic dogs and consisted of a tube-shaped device with a warmed bladder (Amantea 1914; Fig. 1). Amantea’s (1914) AV design was modified for domestic artiodactyls and perissodactyls. By the 1930s, AVs for domestic livestock species were being widely developed (Cole & Winters 1939). Throughout the 1930s–1950s, AV designs for domestic artiodactyls and perissodactyls were modified to suit species-specific penis sizes and ejaculatory cues (Foote 2002). During this time, multiple models of AVs were developed for stallions, including the Colorado model (McCue 2021), the Missouri model (Dascanio 2014), and the Hannover model (Sitters 2014). The Missouri and Hannover model AVs have remained the most frequently used AV models for perissodactyl studies (Dascanio 2014, Sitters 2014). AV use expanded to domestic carnivores and lagomorpha during the 1950s–1970s (Harrop 1954). AVs developed for anseriformes originated in the late 1970s (Tan 1980). AVs were not developed for exotic species until the 1980s and were initially focused on primates (Gould *et al.* 1985). In the 1990s, AVs were adapted for exotic species including marsupials (Johnston *et al.* 1997), followed by cetaceans in the 2000s (O’Brien *et al.* 2008). IMV Technologies and Minitube were the most frequently cited AV manufacturers, followed by Nasco Education (USA). AVs created by researchers instead of commercial manufacturers were most prevalently reported for lagomorpha (Ola 2016), followed by felids (Ackermann & Lopes 2020), and exotic species (e.g. VandeVoort *et al.* 1993). Non-commercially developed AVs have generated effective and inexpensive semen collection devices accessible to researchers in low-income countries (Ola 2016).

### AV study publication rates

**Figure 2:**
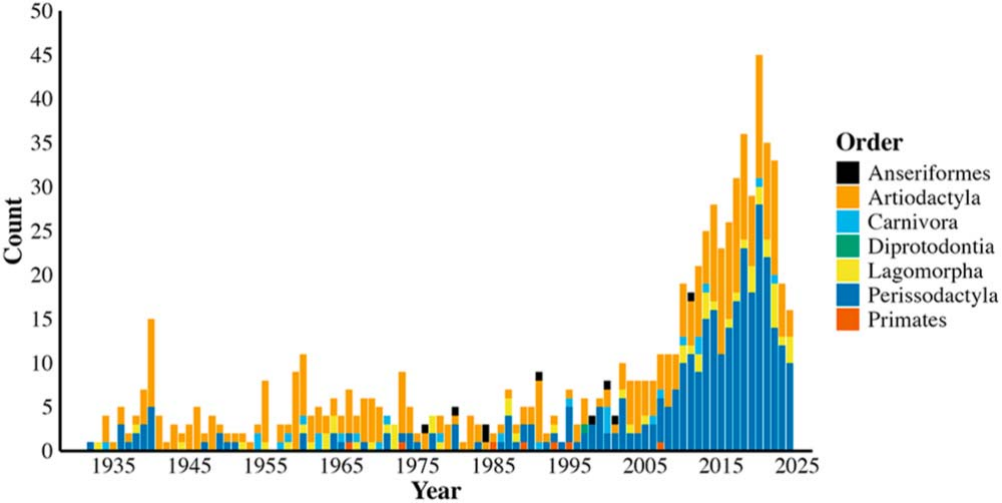
Number of articles published describing AV use in non-human vertebrates between 1914 and 2024. The counts are differentiated by taxonomy (*n* = 778 articles).

The number of publications describing AV use has generally increased since the beginning of the twentieth century. Over the past century, most studies describing AVs focused on domestic perissodactyl species (44% of articles), particularly horses (81% of articles reporting AV use in perissodactyls; Fig. 2). Domestic artiodactyl species have been the next most researched order (43% of articles), with most AVs developed for cattle (38% of articles reporting AV use in artiodactyls), followed by sheep (16% of articles reporting AV use in artiodactyls), and camels (15% of articles reporting AV use in artiodactyls; Fig. 2). Publications on AVs for lagomorpha (6.6%) focused exclusively on domestic rabbits, while those on carnivora (3.7%) were comprised entirely of domestic feline and canine species. AVs designed for anseriformes comprised 1.3% of AV use, and exotic species comprised 1.5% of articles, of which most were designed for primates (67% of articles reporting AV use in exotic species).

### Materials

**Figure 3:**
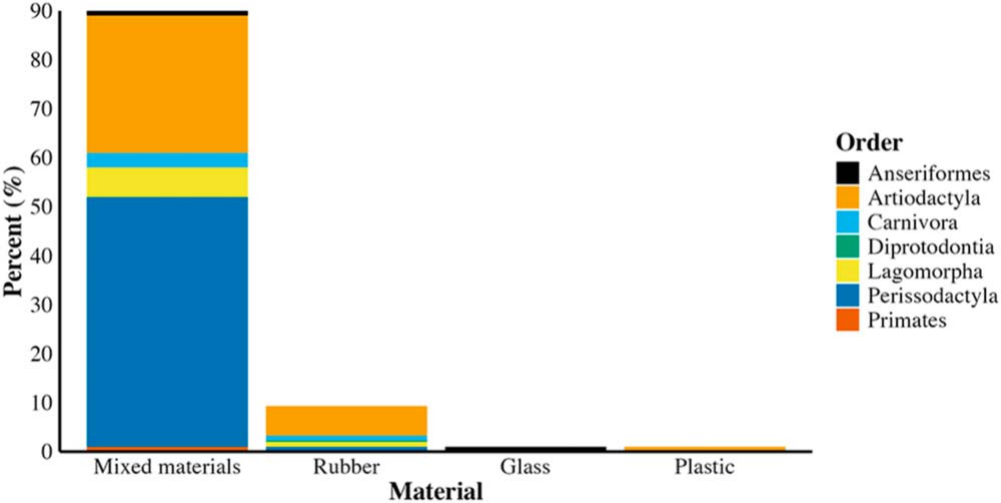
Percentage of AVs constructed with each material differentiated by taxonomy (*n* = 778 articles).

Most AVs were constructed of multiple materials across taxa (Fig. 3), with primarily rubber/latex (47%, Dascanio 2014), leather (39%, Dascanio 2014), and plastic (11%; Ackermann & Lopes 2020) used in mixed-material AVs. Plastic, leather, and rubber are frequently used to create the AV body (the outer rigid structure of the AV), with leather and rubber AV bodies reducing AV weight and improving ease of use compared to plastic AV bodies (Dascanio 2014). Rubber and latex are often used to create the internal AV liner and collection cone (Dascanio 2014). Other materials used in mixed-material AVs included metal (1%, Cruz *et al.* 2011), polyvinyl chloride (0.33%, Bravo *et al.* 1997), silicone (0.33%, Monaco *et al.* 2018), and foam (0.33%, Bravo *et al.* 1997). Mixed-material AVs were particularly prevalent for perissodactyls; different AV models composed of multiple materials were widely adopted for stallions (Love 1992). For example, leather and latex are key components of the Missouri model stallion AV (Dascanio 2014). Although mixed-material AVs were also prevalent for artiodactyls, some AVs for rams (Romano & Christians 2009) and dromedary camels (Cholakkal *et al.* 2016) were comprised exclusively of rubber material. Exclusive use of glass was limited to anseriformes (waterfowl such as geese, ducks, and swans), as penile stimulation does not appear to be necessary to induce ejaculation (Kasai *et al.* 2001). AVs composed exclusively of plastic were limited to artiodactyls, specifically cattle, and had plastic outer structures and liners (Macpherson & Cevallos 1963). The materials used in AV construction did not change significantly over time ($X_{\left(9\right)}^{2}$ = 324.22, *P* = 1, *n* = 778 articles; Supplementary Table 2).

The inner liner of the AV (the portion that directly contacts the penis; Fig. 1) and the collection cone (the portion that connects the body of the AV to the collection vessel; Fig. 1) are most often made of rubber and latex (68%), despite the negative impacts of rubber on sperm reported for multiple mammalian species (Flick & Merilan 1988, Colborn *et al.* 1990, England & Allen 1992; Supplementary Table 1). Textured or smooth latex liners with twists or wrinkles were introduced particularly among artiodactyls, such as camels and cattle, to reflect the variable textures of female reproductive tissues (Vilakazi 2003, Cholakkal *et al.* 2016). Among articles that provided information on the type of inner liner used, 4% specified whether the liner material was textured or smooth. Silicone was cited as an AV liner in 3.5% of articles and was primarily used in AVs for camels and rabbits (Cholakkal *et al.* 2016, Asibor *et al.* 2022) and seemed to have no adverse impacts on sperm properties (Monaco *et al.* 2018). Neoprene has been used as an alternative liner material for AVs in 2% of articles, particularly among species with fibroelastic penises (e.g. buffalos (*Bubalus bubalis*), Baştan *et al.* 2024). Lubricants, such as sterile petroleum jelly, were applied to the liners of many AVs prior to use to reduce the potential for injury to the penis during collection. Some AV liner materials, such as neoprene, do not always require lubricant for species with fibroelastic penises (e.g. cattle and buffalos; Baştan *et al.* 2024). Plastic disposable AV liners and collection cones were reported in 29% of articles and have been favored since the end of the twentieth century because of their reduced disease transmission. Plastic disposable AV liners and collection cones have been widely used on stallions (Alvarenga *et al.* 2016) but may limit semen collection success rates in individuals aversive to the texture (Alvarenga *et al.* 2016).

### Shapes

**Figure 4:**
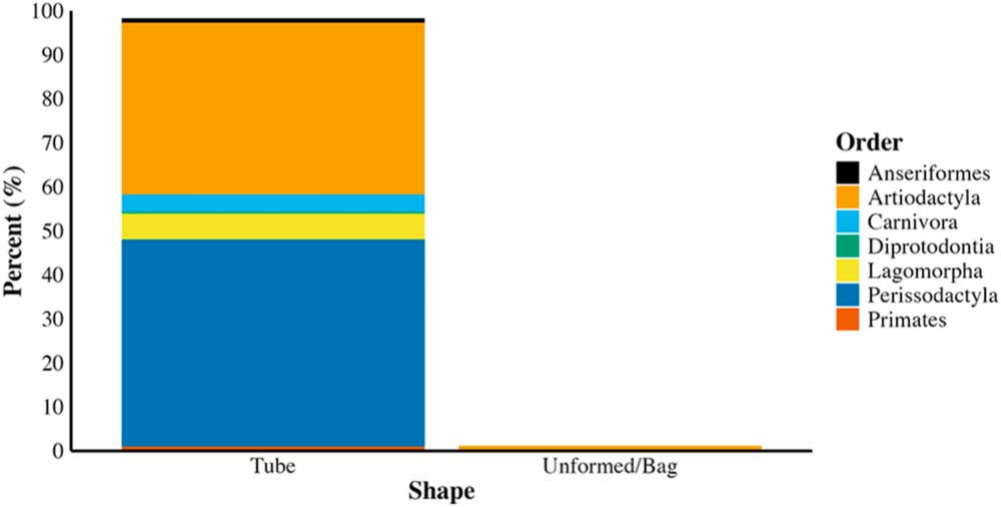
Percentage of AVs of each shape differentiated by taxonomy (*n* = 778).

The majority of AVs were tube shaped (99%; Fig. 4), representing the general shape of the vaginal canal in most vertebrates (Keeffe & Brennan 2023). The few unformed/bag-shaped AVs (1%) were used exclusively with carnivora and artiodactyls, including exotic species such as beluga whales (O’Brien *et al.* 2008) and domestic species such as camels (Mansour 2023). The unformed shape was beneficial as an intravaginal AV for domestic species that conformed to the vaginal lumen shape (Mansour 2023). No article included in the analyses described biological system-shaped AVs (AVs modeled to species-specific vaginal lumen morphologies). Semen collection cone shapes were consistently symmetrical or lacking specific shapes across taxonomic orders. The exception was AVs for perissodactyls, for which asymmetrical collection cones were often used, particularly within the Missouri model AV developed for stallions (Fig. 1; e.g. Sitters 2014). Among symmetrical semen collection cones, funnel-shaped cones (e.g. Aamdal 1960) were more prevalent across all species than tube-shaped cones (e.g. Alvarez *et al.* 2012). The lengths of collection cones were modified concurrently with changes to AV lengths, which varied based on species-specific penis lengths. Among artiodactyls, collection cones were shortened and symmetrical to improve ease of operation, reduce semen contamination, and improve semen quality (e.g. Al-Eknah *et al.* 2001). AVs lacking collection cones (the semen collection vessel was connected directly to the distal portion of the AV body, or the AV body also served as the semen collection vessel) were utilized primarily with felids and lagomorpha (Fig. 1; Andrade *et al.* 2002, Ackermann & Lopes 2020). The shape of AVs did not change significantly over time ($X_{\left(2\right)}^{2}$ = 112.46, *P* = 1, *n* = 778 articles; Supplementary Table 2).

### Elasticities

**Figure 5:**
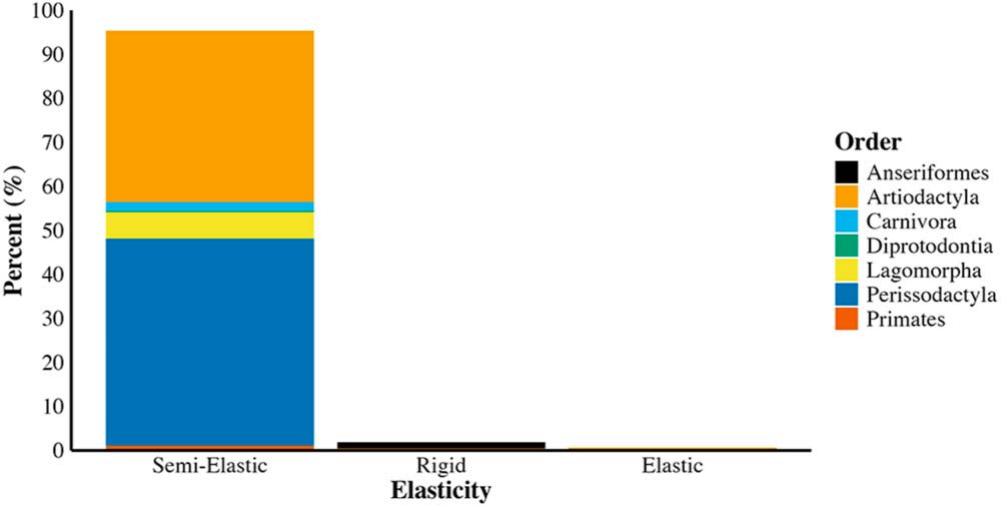
Percentage of AVs of each elasticity differentiated by taxonomy (*n* = 778 articles).

Most AVs were semi-elastic (95.81%; Fig. 5), consisting of a rigid outer shell with a semi-elastic inner liner. The prevalence of semi-elastic AVs is likely due to the widespread use of warmed bladders, which require a semi-elastic construction (Love 1992). Rigid AVs (4.24%; Fig. 1) were created for several mammalian orders, most frequently felids, and few had design augmentations (Tanaka *et al.* 2000, Ackermann & Lopes 2020). Anseriformes were the only order with exclusively rigid AVs and exclusive use of glass (Gvaryahu *et al.* 1984). It has not been tested if alternative AV compositions improve anseriformes’ semen quality compared to rigid glass AVs. Elastic AVs (0.001%) have been used on artiodactyls (camels; Mansour 2023), carnivora (dogs; Otite 2012), and perissodactyls (horse; Ellery 1971). The elasticity of AVs did not change significantly over time ($X_{\left(3\right)}^{2}$ = 223.17, *P* = 0.9959, *n* = 778 articles; Supplementary Table 2).

### Design augmentations

**Figure 6:**
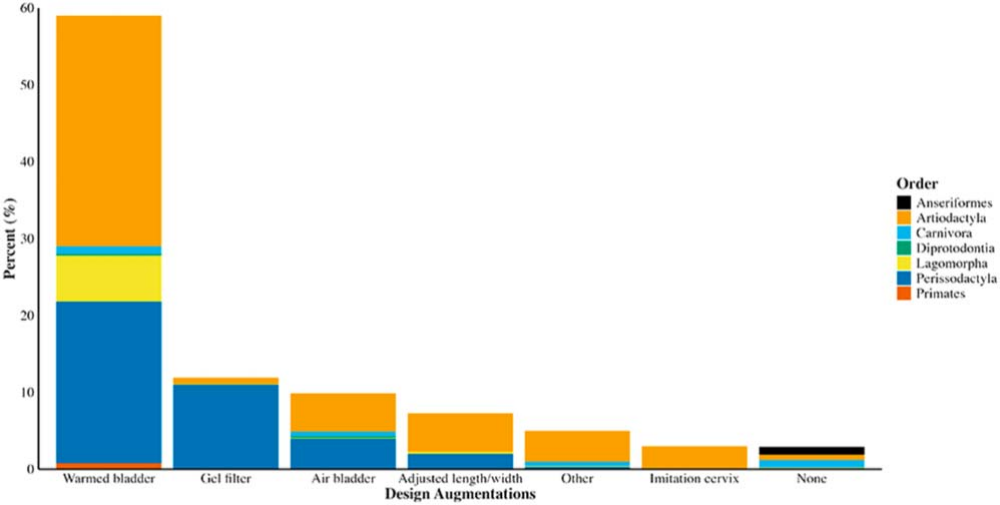
Percentage of AVs with each design augmentation differentiated by taxonomy (*n* = 778 articles).

AV design augmentations were broad within and across taxonomic groups (Fig. 6). Warmed bladders were the most prevalent (59%) and were present in at least one AV for each mammalian order. Warmed bladders stimulate the penis through heat and pressure by filling water at species-specific temperatures in the space between the outer rigid tube and inner liner in semi-elastic AVs (Love 1992). AV internal temperatures varied across taxonomic orders, with internal temperatures between 38 and 45°C for most species of artiodactyl, carnivora, and perissodactyl, and between 34 and 60°C for lagomorpha. Although some AVs possessed an embedded digital thermometer to monitor AV internal temperature (Lichtenwalner 1995), most internal temperatures were measured or estimated with an external device (Al-Bulushi *et al.* 2019). Modifications to warmed bladders that reduced the volume of water in the AV reduced AV weight and improved ease of use (Dascanio 2014). Some AVs (10%) possessed an additional space between the outer tube and inner liner that can be filled with air, creating an air bladder that can be controlled with an external pump (Harrop 1954). Air bladders were adopted for several mammalian orders including artiodactyls and carnivora, particularly among species for which vaginal muscle contractions are known to stimulate the penis during copulation (e.g. pigs; Aamdal *et al.* 1958). Although few articles measured the specific internal AV pressure, several AV designs utilized valves for water and/or air filling that facilitated manual or automatic adjustment of the internal AV pressure based on an individual animal’s penis size (e.g. Aamdal 1960). Modifications to the lengths/widths of commercially available bull/stallion AVs based on species-specific penis lengths/widths were most common among artiodactyls (7%) and improved semen quality through tactile stimulation cues. For example, shortened AVs produced higher quality ejaculates in camels compared to typical length bovine AVs (Cholakkal *et al.* 2016). As the duration of contact with the AV was relatively short for most species (<6 min), tactile stimulation design modifications of AVs were most prevalent for camelid species (e.g. dromedary camels and llamas), which had the longest duration of contact with AVs (5–50 min; Wani *et al.* 2011). Orangutans (*Pongo pygmaeus*) also had a long duration of penile contact with AVs (8–21 min), but no tactile stimulation design modifications were noted for this species (VandeVoort *et al.* 1993). AVs without design augmentations (3%) were developed for carnivora, artiodactyls, anseriformes, and lagomorpha. Anseriformes were the only order for which all AVs lacked design augmentations.

The least common design augmentations were categorized as ‘other’ and included external heating devices (1%), external insulation (1%), external air pumps (0.7%), and modifications to the AV external appearance (0.001%). The use of external insulation and addition of external heating devices to AV semen collection vessels have improved semen quality by reducing cold shock to sperm upon collection for camelid species with long copulation durations (Ferré *et al.* 2015, Wani *et al.* 2011). External air pumps are often utilized in species with extended copulation durations, such as dogs and pigs, to provide additional stimulation (Harrop 1954, Aamdal 1960). Modifying AVs’ external appearance has improved semen collection in species with stimulatory cues involving changes in the genital appearance of females. For example, ovulation in chimpanzees (*Pan troglodytes*) is accompanied by swelling and reddening of the vulva (Graham *et al.* 1973). Incorporating foam that reflects the shape and color of vulval swelling into chimpanzee AVs improved semen collection (Kirsch *et al.* 2022).

Gel filters (12%) have been incorporated into AVs for perissodactyl (e.g. stallions; Ellery 1971) and artiodactyl (e.g. boars; Aamdal *et al.* 1958) species to separate the sperm-rich fraction of semen from the gel fraction. Gel filters are unnecessary for mammalian species that do not produce gel fractions within their semen (e.g. camelids and ovids; Kershaw-Young & Maxwell 2012). Imitation cervixes (3%) were an important design augmentation for camelid species, as interactions between the penis and cervix are important in inducing ejaculation (Bravo *et al.* 1997). Inclusion of an imitation cervix in camelid AVs improved semen quality and copulatory behaviors over AVs without the cervix (Bravo *et al.* 1997; but see Cholakkal *et al.* 2016). Recently, an intravaginal camelid AV that forms to the shape of the vaginal canal collected higher quality ejaculate than other camelid AVs (Mansour 2023), suggesting the female reproductive tract morphology may stimulate the penis and affect semen quality. Despite the differences in design augmentations recorded, the type and prevalence of AV design augmentations did not change significantly over time ($X_{\left(38\right)}^{2}$ = 1,192.3, *p* = 1, *n* = 778 articles; Supplementary Table 2).

## Limitations of AVs

AVs possess limitations as a semen collection technique. Semen collection using AVs is a trained behavior that requires days to months to learn, limiting AV use to domestic/laboratory and captive exotic species that can be safely and effectively trained (Ambrosi *et al.* 2018, Bruno 2022). Animals that can be trained may also refuse to use an AV (Ambrosi *et al.* 2018). In several livestock species, individuals who refused to use an AV had lower libidos, which appear to be a heritable trait (Barth *et al.* 2004). Stimulation to promote arousal prior to semen collection is required for most species (e.g. Wani *et al.* 2011), necessitating the use of a female in natural or induced estrus or her urine. Although changes in AV materials and designs such as cone lengths have improved ease of use and simultaneously reduced collection-associated risks, AV collection poses variable levels of risk of disease transmission and injury to the person collecting semen (Anciuti *et al.* 2025). The use of phantoms/dummy mounts (devices designed to mimic the hindquarters of an animal) combined with specially designed semen collecting locations can reduce contact time between animals and handlers, reducing collection-associated risks (Lindeberg *et al.* 1999, Al-Eknah *et al.* 2001). Proper sanitization, sterilization, and wearing personal protective equipment should be implemented during semen collection from animals, regardless of collection method, to reduce risks to humans. Reusable AV liners and collection cones must be cleaned and sterilized between each use, regardless of material, to reduce disease transmission between animals (Alvarenga *et al.* 2016). Despite the limitations, AVs have been increasingly used for semen collection across taxa since the early 1900s as AVs collect higher-quality ejaculates and promote natural copulatory behaviors compared to other collection methods (Austin *et al.* 1961, Aral & Aral 2004, Ledesma *et al.* 2014). Alternative semen collection techniques, such as electroejaculation and microsurgical aspiration, remain viable options for individuals or species for which AV desensitization and training is not feasible or is unsuccessful. The limitations to AV use and alternative semen collection techniques underscore the necessity to select methods based on taxa-specific reproductive biology and individual animal behaviors and needs.

## Future perspectives

Early AVs were constructed for ease of use by human operators rather than the reproductive anatomy of the target species (Bruno 2022). The introduction of AV design augmentations to account for species-specific stimulatory cues has improved collected semen quality and animal behaviors (Graham *et al.* 1973, Bravo *et al.* 1997). As species differ widely in their reproductive morphologies, copulatory behaviors, and mating systems (Brennan 2016), implementing species-specific AVs that effectively simulate natural copulation may be key to future improvements in ARTs. For example, the development of a bioinspired AV for bottlenose dolphins (*Tursiops* spp.) that incorporated species-specific genital features yielded semen with improved motility parameters compared to manual stimulation collection (Supplementary Fig. 1; Rich *et al.* 2025).

The true prevalence of AV design augmentations and variation of AV compositions may differ from our results due to discrepancies in published AV descriptions and biases in reporting of semen collection methods (e.g. Austin *et al.* 1961). Particularly for artiodactyl and perissodactyl species, AVs have become so common for semen collection that the type of AV utilized is infrequently reported, limiting comparisons across studies. Within taxonomic orders, AV use is reported most often for commercially valuable species. Caution is warranted in assuming species within an order have similarly shaped genitalia, copulatory patterns, or semen quality (Gómez Montoto *et al.* 2011, Jiménez-Rabadán *et al.* 2016, Brennan & Orbach 2020, Orbach *et al.* 2021). We suggest increased reporting of AV models and manufacturers to augment comparisons across studies and elucidate relationships between AV compositions and collected semen quality.

## Supplementary materials







## Declaration of interest

The authors declare that they have no conflicts of interest that could be perceived as prejudicing the impartiality of the research reported.

## Funding

This work was supported by the National Science Foundationhttps://doi.org/10.13039/100000001 (grant #2216595 awarded to DNO).

## Author contribution statement

JR conceptualized the study, collected the data, conducted data analyses and visualizations, and wrote the original manuscript draft. DNO conceptualized and supervised the study and edited the manuscript.
