# Supplementary material for: Artificial vagina conformation and composition for semen collection
Source: Reprod Fertil. 2025 Dec 3;6(4):e250061. doi: 10.1530/RAF-25-0061 (PMC12679965; doi:10.1530/RAF-25-0061)
Supplement: Supplementary file 1 [file supplementary_figure_1.pdf]

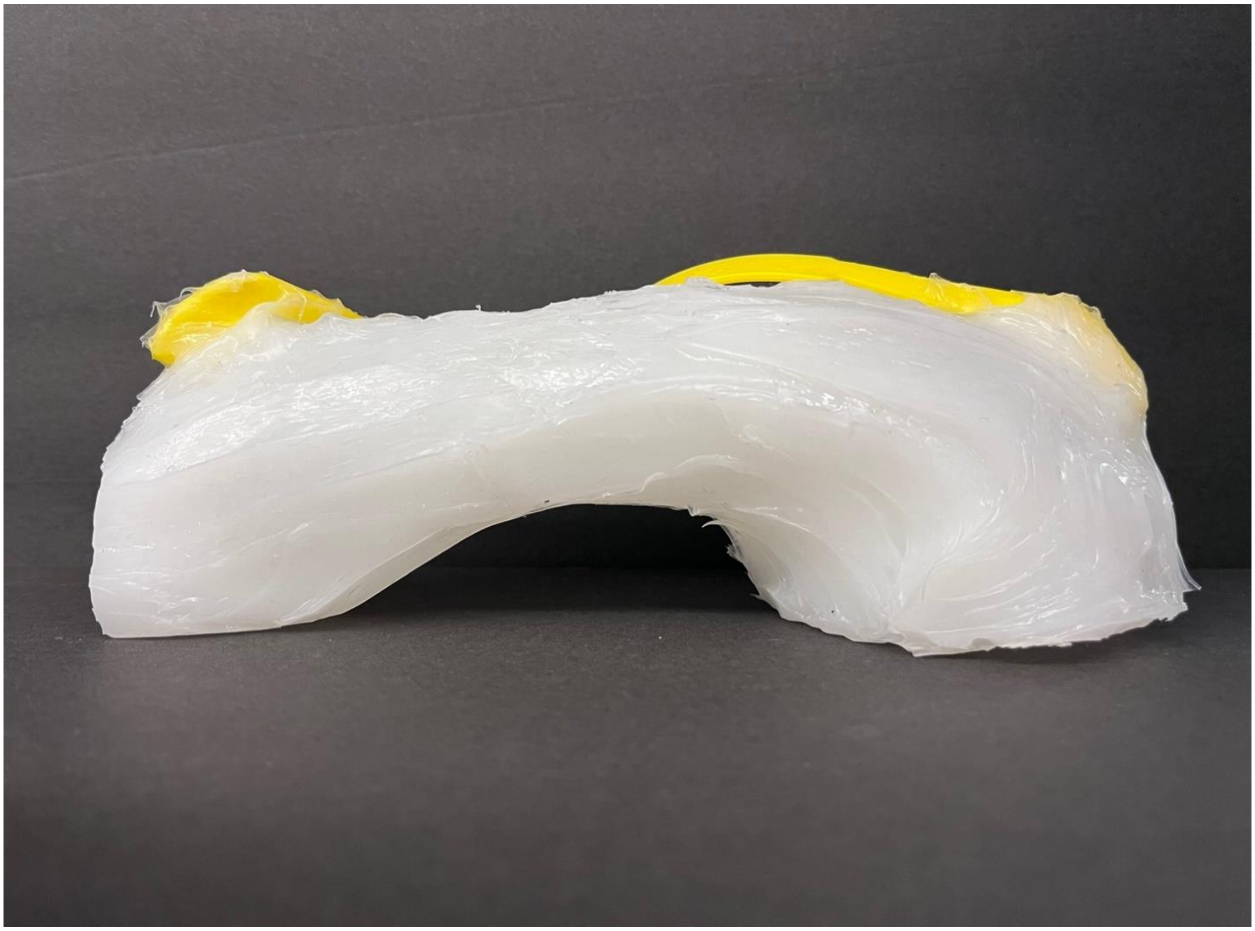

Supplementary Figure 1. Novel bioinspired artificial vagina (BAV) for bottlenose dolphins (*Tursiops* spp.) incorporating species-specific genital morphologies. The BAV is constructed from 3D printed models of dolphin genitalia and created with a silicone possessing a skin-like texture. The right side of the image represents the posterior end of the BAV where the male's penis will enter, while the left side of the image represents the anterior end of the BAV where the semen collection vessel will be attached. The yellow silicone strap on top provides a handle for the operator to hold during collection.
