# Supplementary material for: Artificial vagina conformation and composition for semen collection
Source: Reprod Fertil. 2025 Dec 3;6(4):e250061. doi: 10.1530/RAF-25-0061 (PMC12679965; doi:10.1530/RAF-25-0061)
Supplement: Supplementary file 3 [file supplementary_table_2.pdf]

Supplementary Table 2. Parameter estimates of multinomial logistic regression models assessing temporal trends in artificial vagina compositions.

| Model | Categories | Coefficients | Std error | Odds ratios | p-values | 95% Confidence Intervals |
|---|---|---|---|---|---|---|
| **Material** | Mixed (ref.) | - | - | - | - | - |
| | Glass | - 0.0107 | 2.9097 $e^{-04}$ | 0.9894 | 0.000 | 0.9888 - 0.9900 |
| | Latex | - 0.0003 | 5.0020 $e^{-04}$ | 0.9997 | 0.518 | 0.9987 - 1.0007 |
| | Plastic | 0.0120 | 2.2398 $e^{-04}$ | 1.0121 | 0.000 | 1.0116 - 1.0125 |
| | Rubber | - 0.0156 | 7.2798 $e^{-05}$ | 0.9845 | 0.000 | 0.9844 - 0.9847 |
| | Not provided | - 0.0006 | 4.5575 $e^{-05}$ | 0.9994 | 0.000 | 0.9993 - 0.9995 |
| **Shape** | Tube (ref.) | - | - | - | - | - |
| | Unformed/Bag | 0.0331 | 2.1874 $e^{-04}$ | 1.0336 | 0 | 1.0332 - 1.0341 |
| | Not provided | - 0.0026 | 6.0390 $e^{-05}$ | 0.9974 | 0 | 0.9973 - 0.9975 |
| **Elasticity** | Rigid (ref.) | - | - | - | - | - |
| | Elastic | 0.0146 | 0.0002 | 1.0148 | 0 | 1.0143 - 1.0152 |
| | Semi-Elastic | 0.0015 | 0.0001 | 1.0015 | 0 | 1.0013 - 1.0018 |
| | Not provided | - 0.0057 | 0.0001 | 0.9943 | 0 | 0.9941 - 0.9946 |
| **Design Augmentations** | WB (ref.) | - | - | - | - | - |
| | ALW | - 0.0016 | 1.3654 $e^{-04}$ | 0.9984 | 0.000 | 0.9981 - 0.9986 |
| | ALW, AB, GF, WB | - 0.0023 | 2.5034 $e^{-04}$ | 0.9977 | 0.000 | 0.9973 - 0.9982 |
| | ALW, AB, IC, O, WB | - 0.0029 | 4.9829 $e^{-04}$ | 0.9971 | 3.3409 $e^{-09}$ | 0.9961 - 0.9980 |
| | ALW, AB, IC, WB | - 0.0023 | 2.5075 $e^{-04}$ | 0.9977 | 0.000 | 0.9973 - 0.9982 |
| | ALW, AB, WB | - 0.0021 | 2.0670 $e^{-04}$ | 0.9979 | 0.000 | 0.9975 - 0.9983 |
| | ALW, GF | - 0.0026 | 3.5319 $e^{-04}$ | 0.9974 | 1.7919 $e^{-13}$ | 0.9967 - 0.9981 |
| | ALW, GF, O, WB | - 0.0030 | 5.0148 $e^{-04}$ | 0.9971 | 3.8837 $e^{-09}$ | 0.9961 - 0.9980 |
| | ALW, GF, WB | - 0.0030 | 3.5292 $e^{-04}$ | 0.9974 | 1.7475 $e^{-13}$ | 0.9967 - 0.9981 |
| | ALW, IC, WB | - 0.0021 | 2.2478 $e^{-04}$ | 0.9979 | 0.000 | 0.9974 - 0.9983 |
| | ALW, O | - 0.0024 | 2.9226 $e^{-04}$ | 0.9976 | 2.2204 $e^{-16}$ | 0.9970 - 0.9982 |
| | ALW, WB | - 0.0013 | 9.8527 $e^{-05}$ | 0.9987 | 0.000 | 0.9985 - 0.9989 |
| | AB | - 0.0024 | 2.8969 $e^{-04}$ | 0.9976 | 2.2204 $e^{-16}$ | 0.9970 - 0.9982 |
| | AB, GF | - 0.0026 | 3.5314 $e^{-04}$ | 0.9974 | 1.7852 $e^{-13}$ | 0.9967 - 0.9981 |
| | AB, GF, O, WB | - 0.0024 | 2.9226 $e^{-04}$ | 0.9976 | 2.2204 $e^{-16}$ | 0.9970 - 0.9982 |
| | AB, GF, WB | - 0.0017 | 1.5282 $e^{-04}$ | 0.9983 | 0.000 | 0.9980 - 0.9986 |
| | AB, IC | - 0.0029 | 4.9914 $e^{-04}$ | 0.9971 | 3.4788 $e^{-09}$ | 0.9961 - 0.9980 |
| | AB, IC, WB | - 0.0030 | 5.0727 $e^{-04}$ | 0.9970 | 5.0790 $e^{-09}$ | 0.9960 - 0.9980 |
| | AB, O | - 0.0024 | 2.9206 $e^{-04}$ | 0.9976 | 2.2204 $e^{-16}$ | 0.9970 - 0.9982 |
| | AB, O, WB | - 0.0017 | 1.4712 $e^{-04}$ | 0.9983 | 0.000 | 0.9980 - 0.9986 |
| | AB, WB | - 0.0010 | 7.7087 $e^{-05}$ | 0.9990 | 0.000 | 0.9988 - 0.9991 |
| | GF | - 0.0012 | 8.9383 $e^{-05}$ | 0.9988 | 0.000 | 0.9986 - 0.9990 |
| | GF, WB | - 0.0009 | 6.9063 $e^{-05}$ | 0.9991 | 0.000 | 0.9990 - 0.9992 |
| | IC, O | - 0.0026 | 3.5340 $e^{-04}$ | 0.9974 | 1.8296 $e^{-13}$ | 0.9967 - 0.9981 |
| | IC, O, WB | - 0.0017 | 1.5271 $e^{-04}$ | 0.9983 | 0.000 | 0.9980 - 0.9986 |
| | IC, WB | - 0.0019 | 1.7901 $e^{-04}$ | 0.9981 | 0.000 | 0.9977 - 0.9984 |
| | N | - 0.0012 | 9.5100 $e^{-05}$ | 0.9988 | 0.000 | 0.9986 - 0.9989 |
| | NP | - 0.0007 | 5.6663 $e^{-05}$ | 0.9993 | 0.000 | 0.9992 - 0.9995 |
| | O | - 0.0017 | 1.4742 $e^{-04}$ | 0.9983 | 0.000 | 0.9980 - 0.9986 |
| | O, WB | - 0.0012 | 1.9040 $e^{-04}$ | 0.9980 | 0.000 | 0.9977 - 0.9984 |

ref. = reference category, ALW = adjusted length/width, AB = air bladder, GF = gel filter, IC = imitation cervix, O = other, N = none, NP = not provided, WB = warmed bladder
